# Targeted modulation of gut microbiota by traditional Chinese medicine and natural products for liver disease therapy

**DOI:** 10.3389/fimmu.2023.1086078

**Published:** 2023-02-02

**Authors:** Li-Ran Zhu, Shan-Shan Li, Wan-Qun Zheng, Wei-Jian Ni, Ming Cai, Hai-Peng Liu

**Affiliations:** ^1^ Anhui Institute of Pediatric Research, Anhui Provincial Children’s Hospital, Hefei, Anhui, China; ^2^ Anhui Province Key Laboratory of Medical Physics and Technology, Institute of Health and Medical Technology, Hefei Institutes of Physical Science, Chinese Academy of Sciences, Hefei, Anhui, China; ^3^ Department of Scientific Research and Education, Anhui Provincial Children’s Hospital, Hefei, Anhui, China; ^4^ Department of Chinese Medicine, The First Affiliated Hospital of Anhui Medical University, Hefei, Anhui, China; ^5^ Department of Pharmacy, Anhui Provincial Hospital, The First Affiliated Hospital of USTC, Division of Life Sciences and Medicine, University of Science and Technology of China, Hefei, Anhui, China; ^6^ Inflammation and Immune Mediated Diseases Laboratory of Anhui Province, The Key Laboratory of Anti-inflammatory of Immune Medicines, Ministry of Education, Anhui Institute of Innovative Drugs, School of Pharmacy, Anhui Medical University, Hefei, Anhui, China; ^7^ Department of Pharmacy, Second Affiliated Hospital of Anhui University of Traditional Chinese Medicine, Hefei, Anhui, China; ^8^ Anhui Acupuncture and Moxibustion Clinical Medicine Research Center, Second Affiliated Hospital of Anhui University of Traditional Chinese Medicine, Hefei, Anhui, China

**Keywords:** liver disease, gut microbiota, traditional Chinese medicine, natural product, therapeutic strategy

## Abstract

The gut microbiota not only constitutes intestinal microenvironment homeostasis and human health but also exerts indispensable roles in the occurrence and progression of multiple liver diseases, including alcohol-related liver disease, nonalcoholic fatty liver disease, autoimmune liver disease and liver cancer. Given the therapeutic status of these diseases, their prevention and early therapy are crucial, and the detailed mechanism of gut microbiota in liver disease urgently needs to be explored. Meanwhile, multiple studies have shown that various traditional Chinese medicines, such as Si Miao Formula, Jiangzhi Granules, Liushen Capsules, Chaihu-Shugan Power, Cassiae Semen and Gynostemma, as well as some natural products, including Costunolide, Coprinus comatus polysaccharide, Antarctic krill oil, Oridonin and Berberine, can repair liver injury, improve fatty liver, regulate liver immunity, and even inhibit liver cancer through multiple targets, links, and pathways. Intriguingly, the aforementioned effects demonstrated by these traditional Chinese medicines and natural products have been shown to be closely related to the gut microbiota, directly driving the strategy of traditional Chinese medicines and natural products to regulate the gut microbiota as one of the breakthroughs in the treatment of liver diseases. Based on this, this review comprehensively summarizes and discusses the characteristics, functions and potential mechanisms of these medicines targeting gut microbiota during liver disease treatment. Research on the potential effects on gut microbiota and the regulatory mechanisms of traditional Chinese medicine and natural products provides novel insights and significant references for developing liver disease treatment strategies. In parallel, such explorations will enhance the comprehension of traditional Chinese medicine and natural products modulating gut microbiota during disease treatment, thus facilitating their clinical investigation and application.

## 1 Introduction

Liver diseases are mainly categorized into alcoholic liver disease (ALD), nonalcoholic liver disease (NAFLD), autoimmune liver disease (AILD), liver injury, liver cancer, etc., according to different etiologies and pathogeneses (1). Epidemiological studies have indicated that liver diseases constitute an important part of global morbidity and mortality and have become a huge economic burden and an urgent public health crisis (2, 3). However, due to the lack of understanding of their pathogenesis, late diagnosis and rapid progression, the clinical therapeutic strategies for liver diseases are still insufficient, which directly leads to unsatisfactory treatment effects (4). Therefore, the clinical treatment and management of liver diseases remains a considerable challenge, and there is an urgent need to deeply explore the mechanism and develop promising therapeutic drugs and strategies on this basis.

Increasing evidence has shown that gut microbes are closely associated with the pathogenesis of liver diseases in general (5, 6). A study found an interaction between gut microbiota and the pathogenesis of NAFLD (7), whereas ALD patients exhibited increased intestinal permeability and excessive gut microbiota overgrowth (8). Short-term probiotic supplementation helps restore the beneficial flora in the gut of ALD patients and effectively improves liver function (9). Moreover, dietary cholesterol was found to induce gut microbiota dysbiosis and metabolite alterations in mice that drive NAFLD-hepatocellular carcinoma (HCC) formation, while both cholesterol suppression and gut microbiota modulation showed potential anti-HCC effects (10). Furthermore, gut commensal-controlled bile acid metabolism increases the number of natural killer T (NKT) cells and is related to antitumor immune surveillance of the liver (11). These findings reflect the nonnegligible role of gut microbes in the regulation of liver disease, indicating that more detailed and in-depth mechanistic exploration will provide valuable clues and directions for liver disease therapy.

At present, clinically effective therapeutic regimens and strategies for liver diseases are far from sufficient, which makes it an urgent issue to explore novel drugs and promising therapeutic strategies to overcome the existing deficiencies. Importantly, such a status also facilitates the gradual emergence of hepatoprotective effects of traditional Chinese medicines (TCMs), such as Si Miao Formula, Jiangzhi Granules, Liushen Capsules, Chaihu-Shugan Power, Cassiae Semen and Gynostemma, as well as some natural products (NPs), including Costunolide, Coprinus comatus polysaccharide, Antarctic krill oil, Oridonin and Berberine, both at the basic exploration and clinical research levels. Among them, some TCMs, such as Huazhi-Rougan Formula, have received increasing attention for their protective effects against NAFLD, and terpenoids are considered to be the main active ingredients (12–14). In addition, many NPs, including fisetin, salidroside and oridonin, have also been proven to modulate various liver injuries by regulating the NOD-like receptor thermal protein domain associated protein 3 (NLRP3) inflammasome, and their specific mechanisms remain to be further explored (15). Moreover, TCMs, such as Curcumae rhizome (16) and Xiaoyaosan (17), have shown excellent anticancer activity during liver cancer treatment. These findings suggest that TCMs and NPs can be valuable sources of drugs for liver disease therapy by virtue of their low toxicity, multiple targets and multiple pathways. More delicately, prior studies have found that the therapeutic effects of TCMs and NPs on liver disease are closely related to their regulation of gut microbes. For example, the active ingredient Poria cocos polysaccharides (PCP) downregulates the nuclear factor kappa-B (NF-Kb)/CCL3/CCR1 axis by regulating gut microbes to prevent nonalcoholic steatohepatitis (NASH) (18); Si Miao Formula (19) and Ophiopogon polysaccharide MDG-1 can alleviate NAFLD by inhibiting the gut microbiota and gut-liver axis (20); and oridonin has also been proven to reduce liver injury by altering gut microbiota and promoting the hepatic urea cycle (21). The combination of these studies highlights the great potential of TCMs and NPs in targeting gut microbiota for the treatment of liver diseases.

**Figure 1:**
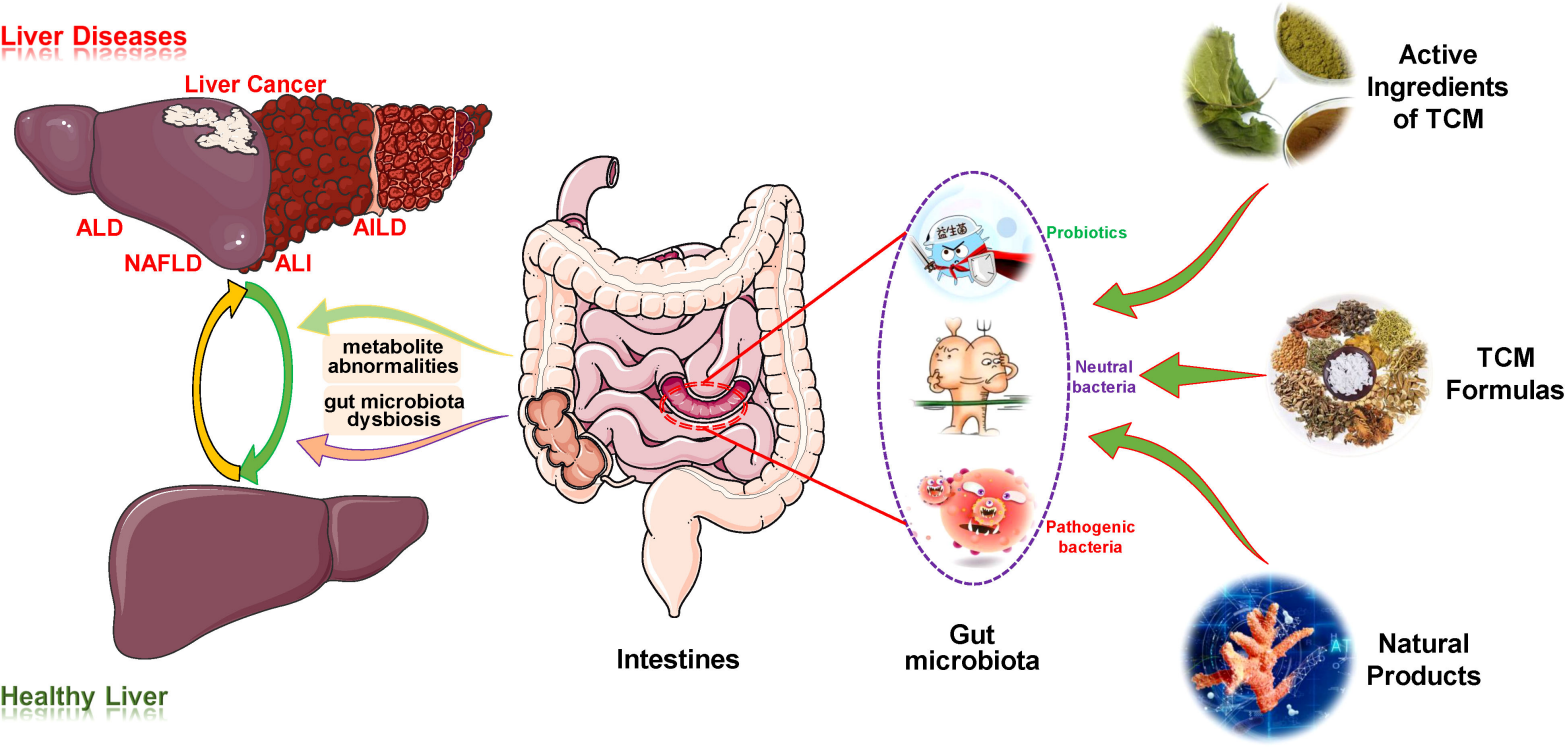
Overview of the therapeutic effects of TCM/NPs targeting the regulation of gut microbiota for liver disease treatment. AIH, autoimmune hepatitis; AILD, autoimmune liver disease; ALD, alcoholic liver disease; ALI, acute liver injury; TCM, traditional Chinese medicine.

Based on the crosstalk between TCMs/NPs, the liver and the gut microbiota, this review comprehensively summarizes and explores the effect and mechanism of such substances in liver disease treatment by targeting the gut microbiota (**Figure 1**). Such exploration provides novel mentality and important references for the establishment of therapeutic strategies for liver diseases and deepens the understanding of these medicines regulating gut microbiota during disease treatment, thus promoting their clinical transformation research and application.

## 2 Targeting gut microbiota for ALD

Studies have confirmed that the progression of ALD is modulated not only by genetic factors, sex, duration and extent of alcohol abuse but also by some potentially modifiable factors, especially the gut microbiota, which provides insights for the mechanistic exploration and therapy of ALD (22, 23).

**Figure 2:**
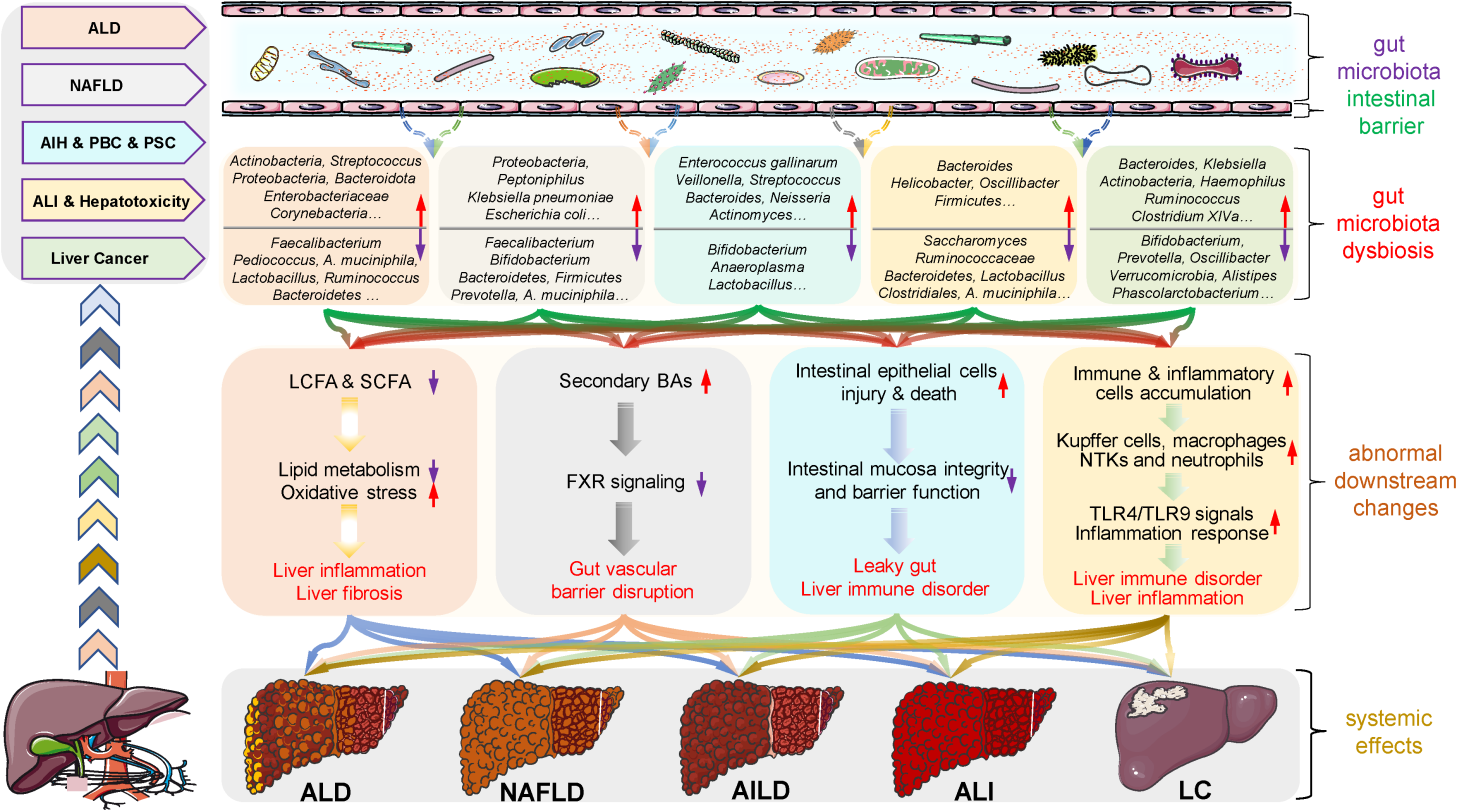
Roles of gut microbiota microenvironment alterations in the occurrence and development of liver diseases. There is growing evidence that gut microbiota dysbiosis is closely associated with the development and progression of several liver diseases, such as alcoholic liver disease (ALD), nonalcoholic fatty liver disease (NAFLD), autoimmune liver disease (AILD), acute liver injury (ALI) and liver cancer (LC). Specifically, alcohol exposure, drugs and other factors induce dysbiosis in the diversity and abundance of gut microbiota species including probiotic, neutrophilic and pathogenic bacteria, as well as abnormal changes in metabolites. These changes subsequently contribute to increased intestinal permeability, intestinal barrier dysfunction, and flora shifts, which in turn accelerate the progression of these diseases through multiple mechanisms, such as the fatty acid metabolic pathway, bile acid (BA) metabolic pathway, inflammatory responses, and immune disorders. These findings reflect the nonnegligible role of gut microbes in the regulation of liver disease and provide valuable clues and directions for the treatment of liver disease. AIH, autoimmune hepatitis; AILD, autoimmune liver disease; ALD, alcoholic liver disease; ALI, acute liver injury; BA, bile acid; FXR, farnesoid X receptor; LC, liver cancer; LCFA, long-chain fatty acid; NAFLD, nonalcoholic fatty liver disease; NTKs, natural killer cells; PBC, primary biliary cholangitis; PSC, primary sclerosing cholangitis; SCFA, short-chain fatty acid; TLR, toll-like receptor.

Alcohol intake or exposure disrupts the ecological balance of the gut microbiota in many ways, and gut microbiota dysbiosis promotes ALD progression through complex mechanisms (**Figure 2**). In this process, alcohol directly leads to the overgrowth and enrichment of some gut microbiota, such as *Actinobacteria*, *Proteobacteria*, *Enterobacteriaceae*, *Corynebacterium* and *Streptococcus*, while the abundance of other gut microbiota subsequently decreases, such as *Bacteroidetes* and *Akkermansia* genera of the phyla *Verrucomicrobia*, *Lactobacillus*, *Ruminococcus*, *Faecalibacterium* and *Roseburia* of the phylum *Firmicutes (* 24). The significant reduction in the number of *Lactobacilli* leads to a reduction in the synthesis of saturated long-chain fatty acids, which in turn accelerates hepatic lipid metabolism, oxidative stress, inflammation and fibrosis, thereby attenuating its protective effect on the liver (25). In addition, acute or long-term drinking leads to injury or even death of intestinal epithelial and immune cells, thus disrupting the integrity and barrier function of the gut mucosa (23). These abnormalities promote some enteric pathogens (especially gram-negative bacteria) and harmful metabolites (lipopolysaccharide (LPS), acetaldehyde, bacterial DNA, and peptidoglycan) to enter the systemic circulation through the gut mucosa, followed by transfer and retention in liver tissue (26). The gut microbiota and harmful substances initiate the downstream immune signal of liver cells through toll-like receptor 4 (TLR4) and other pattern recognition receptors, which not only directly cause an inflammatory response but also enhance the production of inflammatory factors in Kupffer cells/macrophages, thereby aggravating inflammation-induced liver injury (27). Subsequently, inflammatory factors and chemokines gradually accumulate in the hepatic lobules, leading to neutrophil accumulation and further promoting the development of ALD (28).

During ALD treatment, strategies to modulate gut microbiota are gradually being recognized, which is closely related to the nonnegligible effects of TCMs and NPs (**Table 1**). According to prior research, various TCMs and NPs have been found to affect ALD by regulating the ecological balance and function of the gut microbiota. A study showed that probiotic-fermented *Pueraria lobata (Willd.) Ohwi* significantly reduced the abundance of *Bacteroidetes* and *Akkermansia muciniphila* while increasing the abundance of *Firmicutes* and *Lactobacillus*, which modulated aberrant gut microbiota composition and activated nuclear transcription factor (erythroid-derived 2)-like 2 (Nrf2)-mediated signals, thereby improving lipid accumulation and inflammation and exerting antioxidant effects and ultimately preventing ALD (29). One of the naturally occurring sesquiterpene lactones is costunolide, which has been extensively investigated for a wide range of biological activities. A study found that costunolide treats ALD by modulating oxidative stress and reducing inflammation *in vivo* and *in vitro*, which is inseparable from its impact on the gut microbiota (30). Coprinus Comatus polysaccharide, a polysaccharide extract of the edible mushroom species Coprinus Comatus, has been shown to exert prebiotic-like effects to increase gut microbiota diversity, which might improve adverse changes in gut microbiota caused by alcohol consumption and delay ALD progression (31). With extensive research, an increasing number of TCMs, such as Jianpi Liqi Huoxue Decoction (32), Semen Hoveniae Extract (33), and natural products, including Antarctic Krill Oil (34), have been proven to target the regulation of gut microbiota balance, bile acid metabolism and intestinal permeability to treat ALD. Hence, such medicines should be regarded as a valuable source of medicines to delay ALD progression and even achieve therapeutic effects by regulating the abundance, proportion and distribution of gut microbiota, bile acid metabolism and intestinal permeability.

**Table 1:** Research overview of TCMs and NPs targeting gut microbiota for liver disease therapy.

Disease Type	TCM/NP Name	Study Phase	Main Ingredients	Gut Bacterial Alterations	Possible Mechanism	Reference
ALD	Probiotic-fermented Pueraria lobata (Willd.) Ohwi	Preclinical	Polyphenols, Flavonoids, Lactic acids	*Firmicutes/Lactobacillus*↑ *Bacteroidota/Akkermansia↓*	Restore gut microbiota composition, Nrf2 signaling **(+)**, lipid accumulation and inflammation**↓**, antioxidant defense**↑**.	(29)
	Costunolide	Preclinical	Costunolide	*Firmicutes/Actinobacteria↑* *Bacteroidetes/Proteobacteria↓*	Regulate gut microbiota capacities, LPS-TLR4-NF-κB signaling pathway **(-)**, inflammation and oxidative stress**↓**.	(30)
	Coprinus comatus polysaccharide	Preclinical	Coprinus comatus, polysaccharide	*Firmicutes/Muribaculacea/Verrucomicrobia/Lactobacillus*↑ *Rikenellaceae↓*	Increase gut microbiota diversity, gut epithelium barrier integrity damaged **(-)**, SCFA and hepatic gluconeogenesis, insulin resistance and immune responses**↓**, hepatic inflammation and oxidative stress**↓**.	(31)
	Jianpi Liqi Huoxue Decoction	Real World Study	Rhizoma Atractylodis Macrocephatae, Puerariae Lobatae Radix, Radix Paeoniae Alba, etc.	Reverses the abnormal ERIC-PCR fingerprinting of gut microbiota (lacks of specific gut microbials)	Regulates gut microbial abundance/diversity, gut epithelial mucosal permeability**↓**, endotoxin leakage**↓**, reduction of fatty liver and liver injury.	(32)
	Semen Hoveniae Extract	Preclinical	Dihydromyricetin, dihydroquercetin, Quercetin, etc.	*Akkermansia/Verrucomicrobia phylum/Lactobacillus/Alloprevotella/* *Parabacteroides/Bacteroides*↑, *Helicobacter↓*	Alters gut microbial abundance, gut tight junction proteins**↑**, gut epithelium barrier integrity damaged **(-)**, gut leakiness and gut-derived endotoxin absorption**↓**, inflammatory responses**↓**, attenuates hepatic steatosis and NAFLD.	(33)
	Antarctic Krill Oil	Phase II (NCT02089165)	N-3 PUFAs, Astaxanthin, Phospholipids, etc.	*Clostridium Ⅳ/Actinomyces*↑, *Anaerovorax/Methanobrevibacter/Psychrobacter*↓	Regulates gut microbial abundance/diversity, regulate BAs metabolism, BA level**↓**, gut-hepatic FXR/FGF15/FGFR4 axis **(+)**, intrahepatic cholestasis-induced hepatic injury**↓**, attenuates hepatic steatosis and NAFLD.	(34)
NAFLD	Si Miao Formula	Real World Study	Phellodendron chinense Schneid, Atractylodes lancea (Thunb.), Coix lacryma-jobi L. var. mayuen (Roman) Stapf, Achyranthes bidentata BI	*Akkermansia muciniphila*↑	Rebalances gut microbiota composition, gut barrier function ***↑***, lipids metabolism and pro-inflammatory cytokines ***↓***, attenuates hepatic steatosis and NAFLD.	(19)
	Jiangzhi Granules	Phase II (ChiCTR2000034583)	Gynostemma pentaphyllum (Thunb.) Makino, Polygonum cuspidatum Sieb. et Zucc., Salvia miltiorrhiza Bunge., Artemisia capillaris Thunb., Nelumbo nucifera Gaert.	*S24_7/Lachnospiraceae*↑, *Desulfovibrionaceae↓*	Rebalances gut microbiota composition, lipopolysaccharide biosynthesis and sulfur metabolism pathway**↓**, hepatic inflammation level and lipid metabolism**↓**, improves hepatic steatosis, function and insulin resistance, ameliorates NAFLD.	(35)
	Cassiae Semen	Preclinical	Cassiae Semen extract	*Firmicutes/Bacteroidetes/Dehalobacterium/Oscillospira/* *Coprococcus/Ruminococcus*↑, *Erwinia/Klebsiella/Morganella/Trabulsiella/Proteobacteria↓*	Rebalances gut microbiota composition, gut mucosal protein expression**↑**, gut mucosal barrier injury**↓**, endogenous endotoxemia and lipid accumulation**↓**, liver injury and inflammation**↓**, ameliorates NAFLD.	(36)
	Berberine	Phase II (NCT04049396)	Berberine	Bifidobacterium/ Bacteroidetes/Firmicutes↑	Reconstructs gut microbiota composition, tight junction proteins**↑**, regulates BA deconjugation, transformation, and gut barrier function**↑**, bile acid/FXR signaling pathway **(+)**, lipid metabolism/NF-κB activation **(-)**, liver inflammation and oxidative stress**↓**, ameliorates NAFLD.	(37–39)
	Gynostemma	Phase II (NCT05118698)	Gypenosides	*Relative abundance of Bacteroides*↑, *Relative abundance of* *Fissicatena/Akkermansia↓*	Enhances gut microbiota diversity, gut microbiota disorder↓, gut/liver lipid metabolism/insulin resistance↓, gut and liver lesion↓, liver steatosis and lobular inflammation↓, ameliorates NAFLD.	(40)
	MDG-1	Preclinical	Polysaccharide derived from Ophiopogon japonicus	*Akkermansia muciniphila*↑	Increases gut microbial community abundance/diversity, gut barrier function↑, liver lipid accumulation, steatosis and chronic inflammation↓, attenuates NAFLD.	(41)
	Psyllium husk	Preclinical	Psyllium husk	*Sutterella/Faecalibacterium/Coprobacillus/Parabacteroides*↑	Alters gut microbial community, bile acid/FXR signaling pathway **(+)**, serum LPS level↓, hepatic lipid metabolism and NAFLD↓.	(42)
AILD	Liquiritin	Preclinical	Liquiritin	*Bacillus* sp. *46/Veillonella* sp. *31 and* sp. *48/Bacteroides* sp. *22 and* sp. *57/* *Clostridium* sp. *51*↓	Lack of mechanism exploration.	(43)
	Liushen Capsules	Real World Study	Muschus, Artificial Bezoar	*Bifidobacteria/Lactobacillus*↑, *Proteobacteria/Veillonella/* *Prevotella/Neisseria/Actinomyces*↓	Lack of mechanism exploration.	(44)
	Chaihu-Shugan Power	Phase II (NCT03018821)	Bupleurum Falcatum	*Anaeroplasma genus*↑, *Enterobacteriaceae/Staphylococcaceae/* *Veillonella genus*↓	Rebalances gut microbiota composition, NLRP3 inflammasome pathway **(-)**, fat accumulation/inflammatory factor expression↓, chronic metabolic inflammation/AILD/NAFLD↓.	(45)
	GS	Preclinical	Flavonoids, Saponins, Alkaloids	*Lactobacillus/Bifidobacterium*↑, *Streptococcus/Escherichia Shigella/Veillonella/Enterococcus*↓	Rebalances gut microbiota composition, improves gut microenvironment, Nrf2 signaling pathway (**+**), antioxidant activity↑, ameliorates AILD.	(46)
ALI	Oridonin	Preclinical	Oridonin	*Bacteroides vulgatus*↑	Enriches gut *Bacteroides vulgatus*, *Bacteroides vulgatus-*urea cycle-Nrf2 axis **(+)** anti-inflammatory and antioxidative effects↑ balances redox homeostasis against APAP-induced ALI.	(21)
	VTE	Phase II (NCT05052515)	Dihydromyricetin, myricetin, kaempferol, etc.	*Eubacterium_fissicatena group/Ruminococcaceae_UCG-014*↑, *Alistipes/Oscillibacter/Helicobacter*↓	Decreases gut microbiota abundances, tight junction proteins expression↑, intestinal permeability↑ and gut leaky↓, inflammatory response, oxidative stress and abnormal lipid metabolism↓, ameliorates CCl_4_-induced ALI.	(47)
	Zhizichi Decoction	Real World Study	Gardeniae Fructus, Semen Sojae Praeparatum	*Lactobacillus/Romboutsia/* *Akkermansia/Prevotella*↑ *Enterococcus/Parasutterella*↓	Rebalances the gut dysbiosis, butyrate and SCFAs production↑, Keap-Nrf2 signaling pathway **(+)**, oxidative stress↓, host defense against ALI↑.	(48)
	Ganshuang Granules	Early Phase 1 (NCT05523648)	Radix Codonopsis, Bupleurum Falcatum, Salviae Miltiorrhiza	*Lactobacillus/Akkermansia*↑, *Allobaculum*↓	Rebalances the gut dysbiosis, intestinal permeability↓, oxidative stress, inflammatory and hepatic fibrosis↓, ameliorates CCl_4_-induced ALI.	(49)
LC	Shaoyao Ruangan Mixture	Real World Study	Herba Hedyotidis, Scutellariae Barbatae Herba, Paridis Rhizoma, etc.	*Bacteroides*↓	*Bacteroides abundance*↓, IL-10 levels↓, ameliorates unresectable liver cancer.	(50)
	Jiawei Xiaoyao Powder	Real World Study	Radix Angelicae Sinensis, Radix Bupleurum, Radix Codonopsis Pilosulae, Radix Paeoniae Alba, etc.	*Firmicutes/Lachnospiraceae↑*, *Bacteroidetes/Proteobacteria/Bacteroidaceae/Oscillibacter*↓	Regulates gut microbiota Composition and diversity, affects 11 differential metabolites biosynthesis, anti-inflammatory and immunomodulatory effect↑.	(51)
	Panax ginseng	Phase 1 (NCT03775837)	Ginsenosides, Golysaccharides	*Coprococcus/Dehalobacterium/Anaerotruncus/Ruminococcus↑*, *Bacteroides/Arthromitus/Prevotella↓*	Regulates gut microbiota composition and diversity, SCFAs and secondary BAs biosynthesis↑, chronic inflammatory response↓.	(52)
	Safflower yellow	Preclinical	Safflower yellow	*Alloprevotella/Ruminococcus/* *Barnesiella/Bacteroides* *Ersipelotrichaceae incertae sedis↓*	Regulates gut microbiota composition, CD8^+^ T-cell and Gr-1^+^ macrophage mediated immune suppression↑, TNF-α and NF-κB-mediated inflammation↓, regulates tumor immune microenvironment.	(53)
	Zn (II)-curcumin solid dispersion	Preclinical	Polyvinylpyrrolidone (PVP-k30)-based solid dispersion of Zn (II)-curcumin	*Bacteroidetes/Barnesiella/* *Paraprevotella/Prevotella↑*, *Firmicutes/Lachnospiraceae/* *Clostridium XIVa/Oscillibacter↓*	Modulates gut microbiota composition, propionate, SCFA and NKT production↑, liver cancer growth↓, immunotherapy response and efficacy↑.	(54)

AILD, autoimmune liver disease; ALI, acute liver injury; BA, bile acid; CCl_4_, carbon tetrachloride; FGF15, fibroblast growth factor 15; FGFR4, fibroblast growth factor receptor 4; FXR, farnesoid x receptor; GS, ginseng and the seeds of Zizyphus jujuba var. spinosa; IL-10, interleukin-10; LC, liver cancer; LPS, lipopolysaccharide; MDG-1, an ophiopogon polysaccharide; N-3 PUFAs, omega-3 polyunsaturated fatty acids; NAFLD, nonalcoholic liver disease; NF-κB, nuclear factor kappa-B; NKT, natural killer T cells; NP, natural product; Nrf2, nuclear transcription factor (erythroid-derived 2)-like 2; SCFA, short-chain fatty acid; TCM, traditional Chinese medicine; TLR4, toll-like receptor 4; TNF-α, tumor necrosis factor α; VTE, Ampelopsis grossedentata.

**(+)** represents the activation of the signal/signaling pathway**; (-)** represents the suppression of the signal/signaling pathway; “↑” indicates an increase in content, level, or expression; “↓” indicates a decrease in content, level, or expression.

The therapeutic effects of traditional Chinese medicine (TCM) and natural products (NPs) on liver diseases are expected to be inextricably linked to the targeted regulation of gut microbial dysbiosis. Among them, it has been shown that various TCMs, such as Si Miao Formula, Jianpi Liqi Huoxue Decoction, Zhizichi Decoction, Jiangzhi Granules, Panax ginseng and Gynostemma, can affect multiple liver diseases, including alcoholic liver disease (ALD), nonalcoholic fatty liver disease (NAFLD), autoimmune liver disease (AILD), acute liver injury (ALI) and liver cancer (LC). Meanwhile, a variety of active ingredients of TCM, such as Berberine, Gynostemma saponin, Ophiopogon polysaccharide MDG-1, Semen hoveniae extract, and Safflower yellow, can also be used to prevent and treat liver diseases by regulating gut microbial dysbiosis. Furthermore, some NPs, such as Vine tea extract, Costunolide, Antarctic krill oil, and Oridonin, may alleviate or mitigate liver disease by inhibiting the gut microbiota and the gut-liver axis and promoting hepatic urea cycling, among other mechanisms. The table highlights the great potential of TCM and NPs targeting the gut microbiota during the treatment of liver disease.

Although research on the treatment of ALD with these agents targeting gut microbiota has gradually deepened from efficacy observation to mechanism exploration, there are still many deficiencies in the breadth and depth of the current research, such as narrow research scope, unclear target and the lack of clinical trials. Therefore, we need to perfectly integrate existing research with advanced concepts, such as high-throughput screening based on artificial intelligence, proteomics combined with network pharmacology, and research based on clinical phenomena and alterations, which are represented in **Table 1**. These measures will be conducive to more accurate screening of promising therapeutic targets for ALD, form a wider range of potential TCMs and NPs, and establish a deeper target identification system between these drug candidates and ALD-specific targets, which will provide an important reference and novel direction for research on drugs targeting gut microbiota during ALD therapy.

## 3 Targeting gut microbiota for NAFLD

NAFLD is a major cause of chronic liver disease worldwide and is prone to developing into liver fibrosis, cirrhosis and even HCC (55). Although the prevalence rate is increasing yearly, there is still a lack of an ideal treatment method, which makes the exploration of in-depth mechanisms and targeted therapeutic strategies necessary (56).

The exploration of enterohepatic circulation has confirmed that gut microbiota dysbiosis is closely related to the progression of NAFLD, which not only manifested in the great changes in the gut microbiota diversity and abundance in NAFLD patients compared with healthy subjects but also reflected that gut microbiota disrupted the inflammatory balance and glucose and lipid metabolism through intestinal metabolites (57), as shown in **Figure 2**. Specifically, gut microbiota dysbiosis, such as increased abundance of *Proteobacteria* and *Actinobacteria* and decreased numbers of *Bacteroidetes*, *Prevotella and Firmicutes phyla*, reduces the expression of tight junction protein genes, directly leading to the impairment of intestinal barrier function, making harmful microbiota and microorganisms pass through the intestinal barrier, stimulating the immune system and inducing immune cell inflammation, and ultimately accelerating NAFLD and liver fibrosis (58, 59). In addition, a dysfunctional gut microbiota produces various metabolites, such as ethanol, short-chain fatty acids, LPS, bile acids, choline, and ammonia (60). The ethanol produced by *Klebsiella pneumoniae* and *Escherichia coli* under anaerobic conditions can increase the activity of the cytochrome P450 2E1 enzyme, resulting in an increase in reactive oxygen species and free radicals, thus leading to oxidative damage and necrosis of liver cells (61). Meanwhile, the accumulated ethanol also stimulates the NF-κB signal to induce tissue damage by impairing intestinal barrier function, leading to an increased LPS concentration in the portal vein and entry into the enterohepatic circulation. LPS not only stimulates Kupffer cells and hepatic stellate cells and induces steatohepatitis but also promotes the release of TNF-α from hepatocyte macrophages and subsequent insulin resistance, accelerating the development of NAFLD (62). Moreover, studies have found that the differences in bile acids may also affect the dynamics of portal circulation, thus influencing hepatic fat accumulation and the progression of NAFLD (63, 64), while choline deficiency is also associated with the reduced production of very low-density lipoprotein in the liver, which leads to intrahepatic triglyceride accumulation as well as the occurrence of NAFLD (65). In addition, ammonia, a marker of hepatic encephalopathy, is thought to contribute to the pathogenesis of NAFLD through different pathways (66). Hence, strategies targeting the gut microbiota to improve or treat NAFLD will provide novel clues and directions for disease treatment.

According to recent reports, numerous TCMs, including Si Miao Formula [composed of Phellodendron chinense Schneid, Atractylodes lancea (Thunb.), Coix lacryma-jobi L. var. mayuen (Roman) Stapf and Achyranthes bidentata BI], Jiang Zhi Granules [A TCM prescription consisting of Gynostemma pentaphyllum (Thunb.) Makino, Polygonum cuspidatum Sieb. et Zucc., Salvia miltiorrhiza Bunge., Artemisia capillaris Thunb., and Nelumbo nucifera Gaert.], and Cassiae Semen, as well as NPs, such as Berberine and Gynostemma, have been reported to improve NAFLD by regulating the gut microbiota, which provides a promising strategy and direction for NAFLD research and therapy (19, 35, 41, 67). A study found that the classic TCM formula, Si Miao Formula, can significantly alter the composition of the gut microbiota, specifically increasing the proportion of *Akkermansia muciniphila*, which can regulate the expression of genes involved in fat synthesis (e.g., decrease liver sterol regulatory element binding protein expression) and different inflammatory markers (e.g., decrease the expression of interleukin-1β (IL-1β) and IL-6 and the activity of alanine transaminase and myeloperoxidase) (19). These actions positively affect the intestinal barrier function of mice and hepatic fat metabolism to reverse the formation of NAFLD. When exploring the therapeutic effect of Cassiae Semen on NAFLD, Cassiae Semen significantly increased the abundance of *Firmicutes* and *Bacteroidetes* while reducing the number of *Proteobacteria*, thereby alleviating gut microbiota dysbiosis. The restored gut microbiota increases the expression of tight junction proteins in intestinal mucosa, such as zonula occludens (ZO-1) and occludin-1, which will repair the damaged gut barrier and reduce metabolic endotoxemia, ultimately improving lipid accumulation, liver injury, inflammation and even NAFLD (36). The well-known alkaloid active ingredient of NPs, berberine, was shown not only to restore the abundance of *Bifidobacterium*, *Bacteroidetes* and *Firmicutes* to reconstruct the gut microbiota composition and increase tight junction proteins, thus enhancing gut barrier function to ameliorate liver inflammation and oxidative stress (37, 38). Meanwhile, the restored gut microbiota also regulates bile acid deconjugation and transformation to promote the expression of intestinal farnesoid X receptor (FXR) and fibroblast growth factor 15 (FGF15) and further inhibits lipogenesis and NF-κB activation in the liver, thereby activating bile acid/FXR signaling to improve hepatic lipid metabolism (39). These findings suggest that Berberine regulates gut dysbiosis and may be a valuable strategy for the treatment of NAFLD. Furthermore, MDG-1, an Ophiopogon polysaccharide, was shown to inhibit NAFLD by regulating the abundance of *Akkermansia muciniphila (* 41). It was found that PCP can prevent the occurrence of NAFLD, which may be related to the regulation of gut microbiota and inhibition of the NF-κB/CCL3/CCR1 axis (18). In addition to the aforementioned medicines, Jiangzhi Granules and Psyllium husk, as shown in **Table 1**, also showed good anti-NAFLD effects by modulating the gut microbiota (35, 40, 42).

An increasing number of TCMs and NPs have been found to alleviate NAFLD *in vitro* and *in vivo*, which is inseparable from their regulatory effects on gut microbiota. These findings not only indicate that targeting the gut microbiota is a therapeutic strategy with great potential to improve NAFLD but also provide important references for the treatment of NAFLD with these drugs based on gut microbiota. However, although multiple TCMs and NPs have shown promising therapeutic effects at both the cellular and animal levels, research on their transformation and clinical application is seriously insufficient, which is represented in **Table 1**. Meanwhile, we have found that most studies depended on 16S rRNA sequencing analysis, but it can provide only limited analytical value for mechanistic studies. Therefore, alternative methods relevant to human disease models and *in vivo* sterile animal model systems, such as metabolomics and macrometabolic transcriptome analysis, need to be further developed to evaluate microbial functions and their effects on host cells and thus explore mechanisms in depth. Additionally, nonhuman primate (NHP) models and animal models obtained from gut stem cell cultures are of great value to examine those hypotheses derived from clinical observations and for the formation of mechanistic and conceptual conclusions, which will accelerate their clinical translation and application. Nevertheless, the relevance of these findings to initial clinical observations must be confirmed.

## 4 Targeting gut microbiota for AILD

AILD mainly includes autoimmune hepatitis (AIH), primary biliary cholangitis (PBC) and primary sclerosing cholangitis (PSC). It is generally believed that multiple factors, such as heredity, immunity, inflammation, infection, and dysbacteriosis, contribute to the progression of AILD. However, as the complex etiology and pathogenesis of the disease have not yet been fully elucidated, there is currently a lack of specific diagnostic criteria and safe and effective drugs (68). Such a situation urgently requires researchers to conduct in-depth exploration to elucidate the pathogenesis of AILD, identify promising therapeutic targets, and provide a powerful impetus for screening ideal drugs and formulating effective therapeutic strategies.

As shown in **Figure 2**, various lines of evidence have linked gut microbiota dysbiosis with barrier autoimmunity and beyond, especially in the setting of AILD (69, 70). The detection of 16S rDNA sequencing in AIH patients showed that the abundance of *Enterococcus gallinarum* in liver tissue was significantly higher than that in healthy individuals. Moreover, the number of *Bifidobacterium* and *Lactobacillus* in their feces was significantly reduced, which induced a decrease in the proportion of *Bifidobacterium* to *Enterococcus (* 71). These alterations lead to intestinal mucosal damage and increased blood endotoxin levels in patients, thus inducing the immune tolerance damage mechanism and exacerbating liver injury in AIH (72). Meanwhile, liver injury of concanavalin A (ConA)-induced AIH can be significantly alleviated, suggesting that the targeted regulation of gut microbiota is beneficial to AIH therapy. Moreover, a prospective randomized controlled clinical study found that the diversity of gut microbiota in PBC patients was significantly lower than that of healthy controls (*P* = 0.03), and 6 months of ursodeoxycholic acid (UDCA) treatment significantly increased the diversity and abundance of gut microbiota, thereby alleviating PBC progression, which indicates a nonnegligible role for modulating gut microbiota in PBC treatment (73). Furthermore, a study on PSC found a unique correlation between gut microbiota and bile acid, which may be involved in the pathogenesis of PSC by affecting bile acid metabolism and the gut microenvironment; meanwhile, the number of *Veillonella* genera increases with the severity of PSC, suggesting the important role of gut microbiota and its metabolites in the prevention and treatment of PSC (74, 75). These results emphasize the nonnegligible role of gut microbiota dysbiosis in the occurrence and progression of AILD through different pathways or mechanisms, indicating that targeted regulation of the abundance and diversity of gut microbiota may provide new directions and clues for future research and treatment of the disease.

In view of the unignorable or noticeable roles of various gut microbiota in the progression of AILD, it is of great significance to explore TCMs and NPs targeting gut microbiota for AILD research and treatment, as shown in **Table 1**. Liquiritin, the active ingredient extracted from licorice, has been found to significantly inhibit the growth of multiple pathogenic bacteria, such as *Bacillus* sp. *46*, *Veillonella* sp. *31* and sp. *48*, *Bacteroides* sp. *22* and sp. *57* and *Clostridium* sp. *51*, while it has little impact on the growth of commensal probiotics (such as *Lactobacillus* and *Bifidobacterium*), which provides valuable evidence for the potential activity of this herb against gut microbiota during AILD treatment (43). A Chinese medicine called “Liushen Capsule”, produced by Lei Yun Shang Pharmaceutical Group Co., Ltd. with Muschus and Artificial Bezoar as the main ingredients was found to significantly alter the diversity and distribution of gut microbiota in healthy volunteers. Specifically, Liushen Capsule significantly increases the abundance of intestinal anaerobic bacteria (such as *Bifidobacterium* and *Lactobacillus*) while reducing the abundance of some intestinal opportunistic pathogenic microbiota (such as *Proteus*, *Veillonella*, *Prevotella*, *Neisseria* and *Actinomyces*) *(* 44). Meanwhile, both *Bifidobacterium* and *Lactobacillus* were found to be significantly reduced in multiple types of AILD, while opportunistic pathogens were significantly elevated. Thus, Liushen Capsule may be considered a promising drug for the treatment of AILD by targeting the regulation of gut microbiota. In exploring the therapeutic efficacy of Chaihu-Shugan Power, a TCM with Bupleurum Falcatum as its main ingredient, in the treatment of NAFLD. Research showed that the drug significantly reduced the abundance of multiple opportunistic pathogenic bacteria, such as the *Enterobacteriaceae*, *Staphylococcaceae* and *Veillonella* genera, and increased the abundance of the *Anaeroplasma* genus, which shows the potential of Chaihu-Shugan Power targeting gut microbiota dysbiosis during the treatment of AILD (45). In addition, the extract of ginseng and the seeds of Zizyphus jujuba var. spinosa (GS) significantly increased the relative abundance of *Lactobacillus* and *Bifidobacterium* and decreased *Streptococcus*, Escherich*ia coli-Shigella*, *Veillonella* and *Enterococcus* in rats, suggesting that GS extract may be a promising AILD therapeutic drug by balancing the structure and diversity of gut microbiota (46).

Presently, research on gut microbiota-focused AILD treatment has progressed but has mainly concentrated on the exploration of antibiotic applications. Therefore, targeting the gut microbiota to explore novel and promising AILD therapeutic strategies remains an urgent clinical issue. Notably, research on the regulation of gut microbiota dysbiosis in diseases by TCMs and NPs is well underway, which will provide a novel direction for targeting the gut microbiota to explore promising drugs and potential therapeutic strategies for AILD. Even though it is highly expected, the existing studies have the following inadequacies: 1. More studies are still at the efficacy observation stage and fail to address the in-depth mechanism; 2. Research results are more based on the exploration of fecal 16S rDNA sequencing rather than on the gut microbiota. However, the composition or function of the fecal microbiome is different from that of the gut microbiota, which should be taken into account. Therefore, further investigations should be designed to clarify the specific types and targets of bacteria located in the gut, to elucidate the detailed mechanisms by which the gut microbiota regulates AILD and to provide an impetus for large-scale screening and clinical studies of promising TCMs and NPs.

## 5 Targeting gut microbiota for acute liver injury

Various factors, such as drugs, toxicants, and viral infections, produce hepatotoxicity and lead to ALI, which severely damages liver function and inevitably damages human health (76). Prior surveys have shown that ALI frequently occurs year by year and is directly responsible for approximately 3% of global mortality (77, 78). Therefore, preventing and eliminating ALI has become an urgent issue to be solved globally. At present, epigenetics, oxidative stress, inflammatory immunity and other pathological mechanisms have been confirmed to be widely involved in the abnormal activities of hepatocytes and inflammatory immune cells and metabolism-induced ALI, and promising targets and potential therapeutic strategies have also been hotly discussed (79). Meanwhile, the role of gut microbiota in ALI has become increasingly prominent and has gradually attracted attention (80). However, the contribution of the existing studies on the gut microbiota to ALI has not been well characterized, which directly leads to the ineffectiveness of strategies targeting the gut microbiota for disease therapy. In view of this, in-depth exploration of the impact of gut microbiota on ALI will provide new ideas and directions for the treatment of such diseases.

Drug hepatotoxicity is the major cause of clinical ALI in many countries, among which acetaminophen (APAP) is widely studied. 16S rRNA sequencing showed that excessive APAP significantly changed the composition and diversity of gut microbiota, including increasing the ratio of *Firmicutes*/*Bacteroidetes* and reducing the abundance of *Proteobacteria*, *Roseburia*, *Lactobacillus*, *Akkermansia muciniphila* and *Saccharomyces cerevisiae (* 81). The increase in the proportion of *Firmicutes*/*Bacteroidetes* exacerbates liver inflammation and immune disorders, while the decrease in the abundance of *Saccharomyces cerevisiae* leads to the accumulation of the gut microbial metabolite 1-phenyl-1,2-propanedione, which participates in APAP-induced ALI by depleting hepatic glutathione levels (82). The reduction in the proportion of gut *Lactobacillus* promotes oxidative stress and inflammatory responses (83), while *Akkermansia muciniphila* has been shown to modulate immune and metabolic functions (84). In addition, ALI caused by commonly used medications, such as tacrine and diclofenac, has also been proven to be closely related to gut microbial dysbiosis (85, 86). These results indicate that gut microbial dysbiosis is an important factor in promoting drug-induced hepatotoxicity and even ALI. Carbon tetrachloride (CCl_4_) is one of the most common toxicants causing ALI. Integrating 16S rRNA sequencing and LC‒MS metabonomic analysis, CCl_4_ caused the dysbiosis of 32 specific gut microbes in 10 phyla, such as significantly reduced levels of *Firmicutes*, *Clostridiales* and *Lactobacillus* and an increased percentage of *Bacteroides*. The reduction in the abundance of gut *Lactobacillus* promotes CCl_4_-induced liver oxidative stress and the inflammatory response (87), while the elevated ratio of *Firmicutes*/*Bacteroidetes* aggravates liver inflammation and immune disorders75. Meanwhile, *Clostridiales* is significantly positively correlated with 3-hydroxybutyric acid, which has been proven to reduce inflammation and liver injury (88). Moreover, ALI caused by toxicants, such as D-galactosamine and cisplatin, has also been proven to be closely related to gut microbial dysbiosis (82, 86). The aforementioned findings suggest that gut microbial dysbiosis accelerates toxicant-induced ALI (**Figure 2**). Based on these studies, it is of great clinical value and practical significance to target gut microbes to reduce hepatotoxicity and ALI caused by various factors.

In view of the multicomponent, multitarget, multipath and mild effects of TCMs and NPs, their rational use has greater advantages in reducing liver toxicity and even treating ALI. Recently, many studies have demonstrated the great potential of such drugs in this field, which is inseparable from their targeted regulation of gut microbiota, as shown in **Table 1**. Oridonin, a liver protective agent derived from *Rabdosia rubescens*, is believed to reduce APAP-induced hepatotoxicity and ALI by regulating the *Bacteroides vulgatus*-urea cycle-Nrf2 axis (21). A study on the extract of Ampelopsis grossedentata (VTE) indicated that it could alleviate CCl_4_-induced hepatotoxicity and ALI by restoring gut microbiota dysbiosis in mice. Specifically, VTE significantly reduced the content of harmful gut microbiota, such as *Helicobacter* and *Oscillibacter*, and increased the abundance of beneficial gut microbiota, such as *Ruminococcaceae_UCG-014* and *Eubacterium_fissicatena*_group. The gradually restored gut microbiota not only reduces liver inflammation and oxidative stress but also enhances the intestinal barrier by promoting the expression of zonula ZO-1, Occludin-1, and Mucin-1 in intestinal tissues and ultimately achieves the effect of reducing hepatotoxicity and ALI (47). When the gut microbiota was depleted, the disappearance of VTE efficacy verified its targeted regulatory mechanism on the gut microbiota. A TCM composed of Gardeniae Fructus and Semen Sojae Praeparatum, named Zhizichi Decoction, was found to reduce liver injury by regulating the gut microbiota population, promoting butyric acid production and activating the antioxidant reaction (48). In addition, Ganshuang Granules [composed of Chinese herbs such as Radix Codonopsis, Bupleurum Falcatum and Salviae Miltiorrhiza] can rebalance the gut microbiota and reduce intestinal permeability, thereby reducing oxidative stress and inflammation and ultimately ameliorating CCl_4_-induced hepatotoxicity and ALI (49). At present, the potential roles of these agents based on gut microbiota in alleviating hepatotoxicity and ALI have been constantly explored, which not only provides a promising target for the prevention and treatment of the disease but also deepens the awareness of the important research value of TCMs and NPs in disease treatment. More importantly, it offers a novel idea and direction for the establishment of hepatotoxicity and ALI therapeutic strategies by targeting gut microbiota dysbiosis.

Although such medicines targeting gut microbiota dysbiosis have great potential in the prevention and treatment of hepatotoxicity and ALI, there are still some deficiencies and challenges, including the following: 1. Most of the existing studies focus on preset animal experiments while ignoring the characteristics of hepatotoxicity and ALI, which are difficult to observe at the early stage and are progressing rapidly. In other words, it is a huge challenge to consider both mild effects and rapid effects. 2. Hepatotoxicity and ALI are heterogeneous due to different influencing factors, which forces us to choose broad-spectrum or specific treatments. 3. How to avoid the hepatotoxicity of some drugs with different properties while paying attention to their liver protective effect. The above issues provide a direction for subsequent research. Regardless of how difficult the road ahead is, therapeutic strategies based on gut microbiota to regulate hepatotoxicity and ALI will be further developed in the future with the continuous deepening of basic research and the extensive development of clinical research, and the role of these potential drugs will be more clearly clarified.

## 6 Targeting gut microbiota for liver cancer

Liver cancer is one of the most common malignant tumors in the world, and its morbidity and mortality increase each year (89). Although surgical ablation combined with novel targeted drugs, such as sorafenib and atezolizumab, has brought some light to liver cancer patients, the limited types and efficacy of drugs still cannot meet the urgent clinical needs since the pathogenesis of the disease is complex and has not yet been clarified, and most patients are diagnosed at an advanced stage (90). Therefore, it is urgent to elucidate the pathogenesis, explore more potential targets, discover promising drugs, and establish effective targeted therapy strategies for liver cancer treatment.

During the process of exploring potential mechanisms and promising targets, multiple studies have focused on the indispensable role of gut microbiota dysbiosis in promoting liver cancer progression (**Figure 2**). Based on the fecal gut microbiome analysis of HCC patients, it was found that the diversity and abundance of gut microbiota were significantly abnormal, mainly manifested in the increase in *Bacteroides* and *Ruminococcus* and the decrease in *Bifidobacterium (* 91). In contrast to patients with liver cirrhosis, early-stage HCC patients had more intestinal *Actinobacteria*, *Bacteroides*, *Klebsiella* and *Haemophilus*, while *Verrucomicrobia*, *Alistipes, Phascolarctobacterium* and *Ruminococcus* decreased significantly (92). These differences indicated that gut microbiota diversity may be a noninvasive biomarker of HCC and demonstrate an integral role in HCC development. Meanwhile, integrating the microbiome and transcriptome found that *Bacteroidetes*, *Lachnospiracea incertaesedis* and *Clostridium XIVa* were enriched in HCC patients, and their changes in the tumor immune microenvironment through serum bile acids may be important factors associated with liver cancer burden and poor clinical outcomes (93). Gut microbiota omics analysis partially explains the pathogenesis of liver cancer and shows the potential to predict its clinical outcome. Subsequently, *C. scindens* and other *Clostridium enterica* species were shown to utilize bile acid as a messenger to control the accumulation of chemokine-dependent hepatic NKT cells and antitumor immunity in the liver, thus protecting against both primary and metastatic liver cancer (11). This study establishes the relationship between gut microbes, their metabolites and liver cancer, which provides new ideas for future liver cancer treatment. Based on the aforementioned explorations, the gut microbiota should be widely recognized as a valuable and potential therapeutic target in the process of liver cancer research and treatment, and corresponding targeted drug screening, research and the establishment of therapeutic strategies should be emphasized.

TCM and NPs have attracted increasing attention in the treatment of liver cancer, which is inseparable from their targeted modulation of gut microbiota (**Table 1**). In the therapeutic exploration of primary liver cancer (PLC), a TCM preparation produced by Zhejiang Cancer Hospital called Shaoyao Ruangan Mixture (SRM) [composed of 19 Chinese herbs including Herba Hedyotidis, Scutellariae Barbatae Herba, Paridis Rhizoma, Tetrastigma hemsleyanum Diels et Gilg, etc.], could significantly reduce the abundance of Bacteroides in the intestine, which was positively correlated with elevated IL-10 levels and liver cancer development (50). SRM modulates *Bacteroides* to treat PLC, providing an important reference for targeting gut microbiota by TCMs for liver cancer treatment. Meanwhile, fecal microbiology combined with 16S rDNA analysis showed that Jiawei Xiaoyao Powder [consisting of Chinese herbs such as Radix Angelicae Sinensis, Radix Bupleurum, Radix Codonopsis Pilosulae, Radix Paeoniae Alba and Radix Paeoniae Lactiflora] significantly altered the composition of gut microbiota and affected the biosynthesis of 11 differential metabolites, such as primary bile acids and interferon-γ, in liver cancer rats, thus achieving the goal of adjuvant therapy for liver cancer (51). This study provides favorable evidence that TCMs and NPs target the gut microbiota for the treatment of liver cancer. In addition, various TCMs and NPs, such as Panax ginseng (52), Safflower yellow (53) and Zn(II)-curcumin solid dispersion (54), showed valuable therapeutic effects on liver cancer, which is inseparable from their targeted modulation of gut microbiota. Based on these findings, TCMs and NPs targeting gut microbiota are promising therapeutic strategies for liver cancer, while in-depth mechanistic and translational studies need to be further explored.

Currently, research on TCMs and NPs targeting gut microbiota for the treatment of liver cancer is in full swing, and the contribution of multiomics studies with high-throughput screening is outstanding. However, the existing studies still have many limitations, mainly including the following: 1. Most studies have focused on exploring the effects of these medicines on the gut microbiota *in vitro*, ignoring the complexity of the real environment of the organism; 2. Most studies have focused on the changes in gut microbiota after the inhibitory effects of those agents on liver cancer but neglected direct evidence of their targeting of gut microbiota; 3. Insufficient sample size directly led to different individuals showing high variability in the composition and abundance of gut microbiota, which limited the generalizability of conclusions regarding gut microbiota. Based on the present situation, subsequent studies should focus on 1. In-depth exploration of pharmacological mechanisms; 2. Research targeting specific gut microbiota; 3. Larger sample sizes are needed to overcome such variability and draw meaningful conclusions; 4. Exploration oriented to the metabolic processes and metabolites of TCMs and NPs.

## 7 Concluding remarks and perspectives

The gut microbiota is essential for maintaining body metabolism and health, while dysbiosis plays a vital role during the occurrence and progression of various diseases, including liver disease (94, 95). Therefore, regulating the gut microbiota to maintain it in a relatively stable state, including gut microbiota diversity, distribution and metabolic stability, has great potential and clinical research value in the treatment of various types of liver diseases. Meanwhile, we should realize that the current research mostly emphasizes the correlation between gut microbiota dysbiosis and disease, as well as disease outcome and gut microbiota alterations, which is far from sufficient. The importance of the gut microbiota is increasingly prominent. However, we must recognize that current research has emphasized the correlation between gut microbiota dysbiosis and disease and the correlation between disease outcome and gut microbiota alteration, which is insufficient. Hence, future research should give more attention to 1. Exploring in depth the detailed mechanisms by which gut microbiota dysbiosis directly contributes to disease pathogenesis; 2. How to develop precision medicine by accurately regulating gut microbiota to implement disease-specific treatments. Based on these findings, it is first necessary to perform in-depth information exploration of bioinformatics resources such as gut microbiomics and metabolomics. Subsequently, the acquired precise information will be explored and validated mechanistically in specific cellular and organoid models. Eventually, the clinical effects will be explored through bacterial colonization and the achievements will be translated. These lines of thought are quite helpful for establishing therapeutic strategies targeting the gut microbiota.

TCMs and NPs provide a huge source for the research and discovery of new drugs by virtue of their obvious anti-inflammatory, antioxidant, liver protective and other effects and have attracted widespread attention. As reported by many researchers, such drugs have played a beneficial role in regulating liver diseases such as ALD, NAFLD, AILD, hepatotoxicity, and liver cancer, which are inseparable from the regulation of gut microbiota dysbiosis (96). However, the majority of existing studies remain at the stage of preliminary pharmacodynamic validation, which makes the development of such drugs and the establishment of therapeutic strategies inadequate. Therefore, future research should emphasize the following: 1. To adequately integrate technical solutions such as artificial intelligence, omics and high-throughput screening to screen and expand the fingerprint profiles and databases of TCMs and NPs to provide resources for the discovery of more drugs; 2. To develop diverse therapeutic routes to meet the broad spectrum of TCMs and NPs to regulate gut microbiota for disease treatment; 3. To advance the isolation techniques of TCMs and NPs together with the improvement of formulation technology and preparation procedures to provide the basis for targeted regulation of gut microbiota therapy rather than wide scattering to cure diseases.

## Author contributions

W-JN, MC and H-PL made substantial contributions to the conception or design of the work. L-RZ, S-SL and W-QZ contributed to the acquisition, analysis, and interpretation of literature for the work. L-RZ, W-JN, MC and H-PL drafted the work or revised it critically for important intellectual content. W-JN, MC and H-PL final approval of the version to be published. All authors agree to be accountable for all aspects of the work in ensuring that questions related to the accuracy or integrity of any part of the work are appropriately investigated and resolved. All authors contributed to the article and approved the submitted version.
